# Toxicological Effects of Lufenuron on Antioxidant Defence in Grass Carp (*Ctenopharyngodon idella*)

**DOI:** 10.1002/vms3.70626

**Published:** 2025-09-29

**Authors:** Maria Saeed Khan, Abdul Ghaffar, Qudrat Ullah, Muhammad Farooq, Habiba Jamil, Muhammad Israr, Ibrahm A. Alhidary

**Affiliations:** ^1^ Department of Zoology The Islamia University of Bahawalpur Bahawalpur Pakistan; ^2^ Department of Theriogenology Cholistan University of Veterinary and Animal Sciences Bahawalpur Pakistan; ^3^ Soil, Plant and Food Science University of Bari Aldo Moro Bari Italy; ^4^ Task Force Health Care Limited Ilford, London UK; ^5^ Department of Animal Production College of Food and Agriculture Sciences King Saud University Riyadh Saudi Arabia

**Keywords:** aquaculture, *Ctenopharyngodon idella*, antioxidant enzymes, lufenuron, toxicity

## Abstract

Lufenuron, an acyl urea insecticide, is widely used in agriculture, but its ecotoxicological impact on freshwater fish remains poorly understood. The present study investigated the biochemical and antioxidant responses of grass carp (*Ctenopharyngodon idella*) following chronic exposure to sublethal concentrations of lufenuron (2–4 µg/L) for 33 days. A total of 60 fish were randomly distributed into control and treatment groups, and tissues, including liver, kidney, gills, heart and brain, were sampled on Days 11, 22 and 33. Antioxidant enzyme activities—superoxide dismutase (SOD), peroxidase (POD), catalase (CAT) and glutathione (GSH)—were quantified spectrophotometrically. Results revealed organ‐specific responses to lufenuron exposure. SOD and GSH levels increased significantly (*p *< 0.05) in all examined tissues, indicating enhanced defence against oxidative stress. In contrast, POD activity declined in the gills and kidney but increased in the liver, brain and heart. CAT activity was elevated in the liver, kidney and gills but decreased markedly in the heart and brain, suggesting tissue‐dependent vulnerability to oxidative damage. Overall, the findings demonstrate that lufenuron induces oxidative stress in *C. idella*, disrupting enzymatic antioxidant defences in detoxifying and non‐detoxifying organs. Antioxidant enzymes are thus reliable biomarkers for assessing the ecological risks of pesticide contamination in aquatic environments.

## Graphical abstract

GRAPHICAL ABSTRACT

Lufenuron exposure in grass carp disrupted antioxidant enzyme activities in a tissue‐specific manner, indicating oxidative stress. Superoxide dismutase and glutathione increased, whereas catalase and peroxidase showed organ‐dependent alterations, highlighting antioxidant enzymes as sensitive biomarkers for assessing ecotoxicological risks of pesticide contamination in aquatic ecosystems.

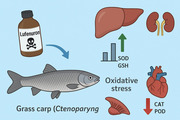

## 1 Introduction

Aquaculture contributes significantly to global food security, as fish and aquatic products serve as a primary protein source in many regions (Kalita et al. 2023; Al‐Mahish et al. 2024; Alsulami et al. 2024). However, freshwater ecosystems—the foundation of aquaculture—are among the most vulnerable to environmental contaminants, particularly pesticides, which reduce biodiversity, cause fish mortality and impair growth, reproduction and tissue integrity (Pisa et al. 2015; Hussain et al. 2024; Mukanga et al. 2024; Oleinikova et al. 2024).

Lufenuron, a benzoylurea pesticide, is widely used in agriculture for pest control due to its specificity, rapid environmental degradation and effectiveness against immature insects (Hafeez et al. 2019). It acts by disrupting insect growth, moulting and chitin synthesis (Vázquez et al. 2014). Despite these advantages, evidence suggests that lufenuron and similar pesticides can adversely affect non‐target aquatic organisms. In fish, pesticide exposure has been associated with haematological disturbances, endocrine disruption, tissue damage and alterations in antioxidant defence mechanisms (Ghelichpour et al. 2020, 2019).

Oxidative stress plays a central role in pesticide‐induced toxicity. Free radicals generated under such stress damage fish physiology, whereas antioxidant enzymes—including superoxide dismutase (SOD), catalase (CAT), glutathione peroxidase (GPx) and peroxidase (POD)—serve as biomarkers of the defence response (Kupradit et al. 2023; Yigit et al. 2024). Variations in their activity depend on exposure level, duration and species susceptibility (Basit et al. 2023). For instance, pesticide stress has been shown to reduce SOD and GPx expression in fish while altering CAT activity in response to environmental conditions (Baag et al. 2021). Such changes are indicative of oxidative imbalance, which is often coupled with increased susceptibility to opportunistic pathogens such as *Aeromonas hydrophila*, a major cause of fish morbidity and mortality (Guo et al. 2019).

Grass carp (*Ctenopharyngodon idella*), an important aquaculture species with global distribution, depends on optimal intestinal and systemic health for growth and productivity (Jia et al. 2024). Given the increasing use of lufenuron and its potential risk to non‐target species, understanding its effects on carp physiology is critical. Therefore, the present study aimed to evaluate the toxicological impacts of lufenuron exposure in grass carp by examining key haematological and biochemical parameters. In particular, we assessed the concentration‐dependent activity of antioxidant enzymes (POD, CAT, glutathione [GSH] and SOD) in liver, kidney, heart, brain and gill tissues to clarify the mechanisms of oxidative stress and antioxidant defence in response to pesticide stress.

## 2 Materials and Methods

### 2.1 Tested Chemical

Lufenuron, recognized as an acyl urea insecticide, is characterized by the chemical formula (1‐[2,5‐dichloro‐4‐(1,1,2,3,3,3‐hexafluoropropoxy)phenyl]‐3‐(2,6difluorobenzoyl) urea) used to examine its effect on enzyme activity and histological alteration in detoxifying organs of freshwater fish grass carp (*C. idella*). The solution was quickly made by dissolving a 5% emulsified lufenuron in distilled water, with a volume of 1 mL for each solution, in order to achieve the necessary concentrations of lufenuron (2–4 µg/L). The experimental applications were then carried out using these solutions.

### 2.2 Experimental Fish

A total of 70 healthy freshwater *C. idella* (weight range: 50–100 g) were procured from the Government Hasilpur Fish Hatchery and transported to the Department of Zoology, The Islamia University of Bahawalpur, in oxygenated plastic bags. On arrival, fish were transferred into glass aquaria (100 L capacity; 40 × 20 in.) and allowed a 10‐day acclimatization period under controlled laboratory conditions. For the experiment, fish were randomly allocated to five treatment groups (including control), with each group replicated across separate 100 L aquaria (*n* = 14 fish per tank). Randomization was carried out by assigning fish to tanks using a random number table to minimize selection bias. The sample size (14 fish per replicate tank) was determined on the basis of previous toxicological studies in cyprinids reporting sufficient statistical power to detect treatment effects, while also adhering to ethical considerations for minimizing animal use.

### 2.3 Physiochemical Parameters

The physiological and chemical properties of the water were measured both before and after the experiment. The water temperature, pH and electrical conductivity of each aquarium were determined with the help of a pH tester (HI98107pHep), an EC tester (HI98304DiST 4) and an electric thermometer. The salinity, total dissolved solids and dissolved oxygen were tested by using a salinity tester (Marine Line/HI98319), a TDS tester (HI98302DiST 2) and a DO meter (DO meter Hanna 2400). The safety of laboratory organisms was maintained according to the guidelines published by the Islamia University of Bahawalpur office of the Directorate of Research and Bioethics committee. Table 1 shows all physicochemical parameters recorded at the start of experimental trial period in aquarium.

**TABLE 1:** The physicochemical parameters recorded at the start of experimental trial period in aquarium.

Physicochemical parameters	Aquarium no. 1 T1	Aquarium no. 2 T2	Aquarium no. 3 T3	Aquarium no. 4 T0
Water temperature (°C)	28	31	32	30.9
Salinity (ppt)	0.4	0.4	0.5	0.3
TDS (ppt)	0.42	0.43	0.44	0.41
EC (µS/cm)	0.80	0.83	0.82	0.77
pH	7.2	7.3	7.4	7.0
DO (ppm)	4.0	4.1	4.3	5.0

### 2.4 Optimal Conditions for Maintenance of Aquariums

The optimal conditions were maintained through continuous air pumps, which provided sufficient oxygen to the exposed fish in all aquariums, whereas the water temperature was carefully maintained. Four aquariums with the size of 30 × 18 × 15 in., having 100‐L water capacity, were used for the study. Oxygen was maintained using an oxygenator (oxygen pump). Each aquarium was appropriately cleaned with a waste‐removing sieve and a wet sponge. Net clothing and proper clips were used to cover the open end of each aquarium to prevent the fish from escaping. Fish were all fed regular fish food that had ample protein (24%). Feed was composed of pellets; feed of 2 kg was given once a week, 4 days a week at 9:00 AM; large fish were fed at 12% of their body weight, whereas small fish were fed at 10% of their body weight. Each aquarium's waste (fish excretions) and leftover feed were filtered and taken out daily.

### 2.5 Experimental Design

After acclimatization, the trial fish (60; weight range: 65–100 g) were divided equally into four experimental groups. Within each group, consisting of 15 fish, random assignment (T1, T2, T3 and T0 control group) was performed, followed by the administration of the insecticide lufenuron. The control group was named group T0, whereas groups T1–T3 were exposed to variable concentrations of lufenuron. The relevant concentration of lufenuron (2–4 µg/L) was employed for research, and the treatment persisted for 33 days. Over this period, samples of diverse tissues, including kidney, gills, heart and liver, were collected from each treated fish within all investigational groups on 11, 22 and 33 days. These tissue samples were preserved in 10% formalin for later analysis. We observed that the fish exposed to lufenuron in three separate groups displayed surface breathing, body effects, instability and delayed neurotic growth.

### 2.6 Biochemical Analysis

Fish were sampled on Days 11, 22 and 33 of the experiment for biochemical assessment. Prior to dissection, fish were euthanized by immersion in an MS‐222 solution (250 mg/L water for 10 min). Internal organs (brain, liver, kidney, heart and gills) were excised, rinsed with distilled water, homogenized in ice‐cold phosphate‐buffered saline and stored in Eppendorf tubes until analysis. Enzymatic activities were determined using a UV–visible spectrophotometer (Libra 752 PC; Biochrome, UK).

### 2.7 Antioxidant Enzyme Activity

The activity of antioxidant enzymes—including SOD, POD, CAT and GSH—was quantified in homogenized tissues of brain, kidney, liver, gills and heart.

### 2.8 SOD Activity

SOD activity was measured following the method of Jindal and Kaur (2014). SOD converts superoxide radicals into hydrogen peroxide, thus serving as a first‐line defence against oxidative stress. A reaction mixture was prepared by combining 1.5 mL methionine, 1 mL nitro blue tetrazole (NBT) and 0.75 mL Triton X‐100, and the volume was made up to 30 mL with PBS. From this, 1 µL of solution and 20 µL of tissue homogenate were added into a centrifuge tube. After incubation at 37°C for 5 min, 10 µL riboflavin was added, followed by further incubation at 40°C for 8 min. Absorbance was measured at 560 nm, with three replicates per sample.

POD activity was determined according to Civello et al. (1995). POD protects cells against excessive accumulation of H_2_O_2_, thereby reducing oxidative stress. A substrate solution was prepared by mixing 50 µL guaiacol with 47 mL phosphate buffer, followed by the addition of 150 µL H_2_O_2_ and 1250 µL PBS. Then, 50 µL of tissue homogenate was added, and absorbance was recorded at 470 nm. Three readings were taken per sample.

CAT catalyses the decomposition of H_2_O_2_ into water and oxygen, thereby protecting cells from oxidative stress. A sodium phosphate buffer of 2 mL was used as a blank (set to zero at 240 nm). For measurement, 1.95 mL of buffer was mixed with 0.05 mL of tissue homogenate. The reaction was monitored for 3 min, and absorbance was recorded at 240 nm.

A reaction mixture was prepared by adding 1 mL disodium hydrogen phosphate buffer, 0.1 mL homogenate sample and 0.5 mL DTNB reagent. Absorbance was measured at 412 nm. Three readings per sample were taken and averaged.

### 2.9 Statistical Analysis

A two‐way analysis of variance (ANOVA) was utilized to calculate the degree of significant change between lufenuron‐treated and untreated fish. All experimental data were presented as mean ± SE, and a significance threshold of *p* > 0.05 was used. Statistical analysis was done with the help of SPSS 15 software. For this purpose, the post hoc Tukey's test was employed to assess variation in mean values (mean ± SE) that present the oxidative stress and antioxidant enzymes in the blood and internal organs of both control and experimental groups. Additionally, a correlation analysis of Pearson was performed to observe the variables in various organs of treatment groups.

## 3 Results

The results showed that lufenuron exposure had a significant effect on enzyme activity in the detoxifying organs of freshwater fish (*C. idella*).

### 3.1 Antioxidant Enzyme Studies

From the gills and liver of fish, cancer prevention agent proteins, such as CAT, GSH, POD and SOD, were isolated to examine biochemical parameters. Organs after dismemberment were homogenized in 0.2 M cold phosphate cushion blend in the proportion of 1:4 (w/v). Homogenated organs were undergoing centrifugation at 4°C and 10,000 rpm for 15 min. After the centrifugation process, unadulterated supernatants were put away at −80°C for the chemical measure, whereas build‐ups were squandered. The SOD action was noted by watching its capacity to stop the photograph decrease of NBT.

### 3.2 Antioxidant Enzymes in Gills

**FIGURE 1:**
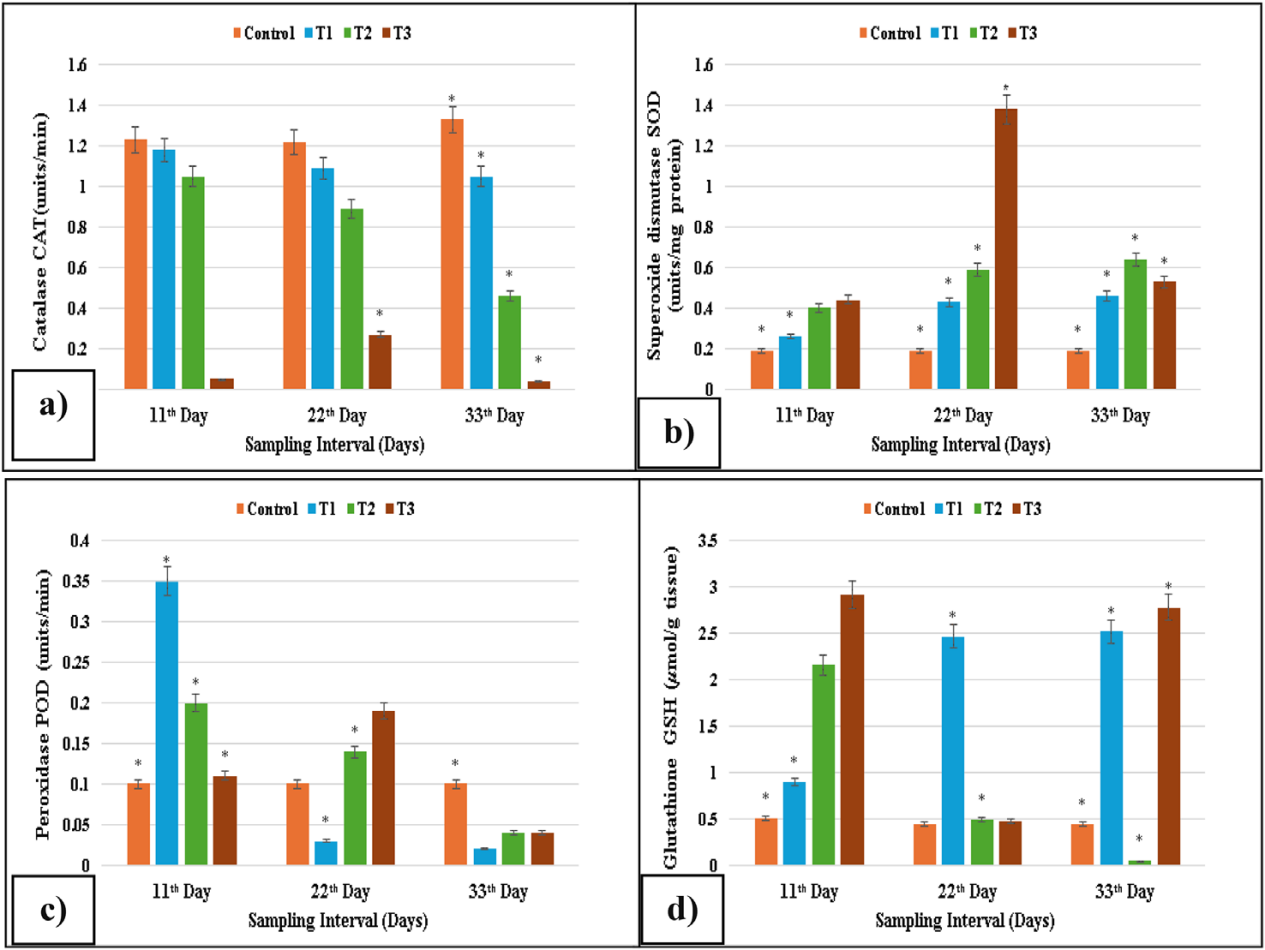
Antioxidant enzyme activity: (a) CAT activity reduced at higher concentrations on Days 11, 22 and 33; (b) SOD activity increased at higher concentrations on Days 22 and 33; (c) POD activity reduced at the maximum concentration on Day 33; (d) GSH activity reduced on Day 22 at higher concentrations, whereas it increased on Day 33 at higher concentrations in isolated cells of gills of lufenuron‐treated and control fish. * indicates significantly different at *p* < 0.05.

The gills of fish treated with different concentrations of lufenuron showed considerable change in the levels of POD, CAT, SOD and GSH activity after 11, 22 and 33 days. From this investigation, it has been shown that SOD and GSH contents of treatment groups are higher as compared to control group, whereas the activity of POD was significantly (*p* < 0.05) lower in the gills of treated fish. However, as compared to control group, the activity of CAT was significantly (*p* > 0.05) enhanced in the gills of exposed *C. Idella*, as shown in Figure 1.

### 3.3 Antioxidant Enzymes in Liver

**FIGURE 2:**
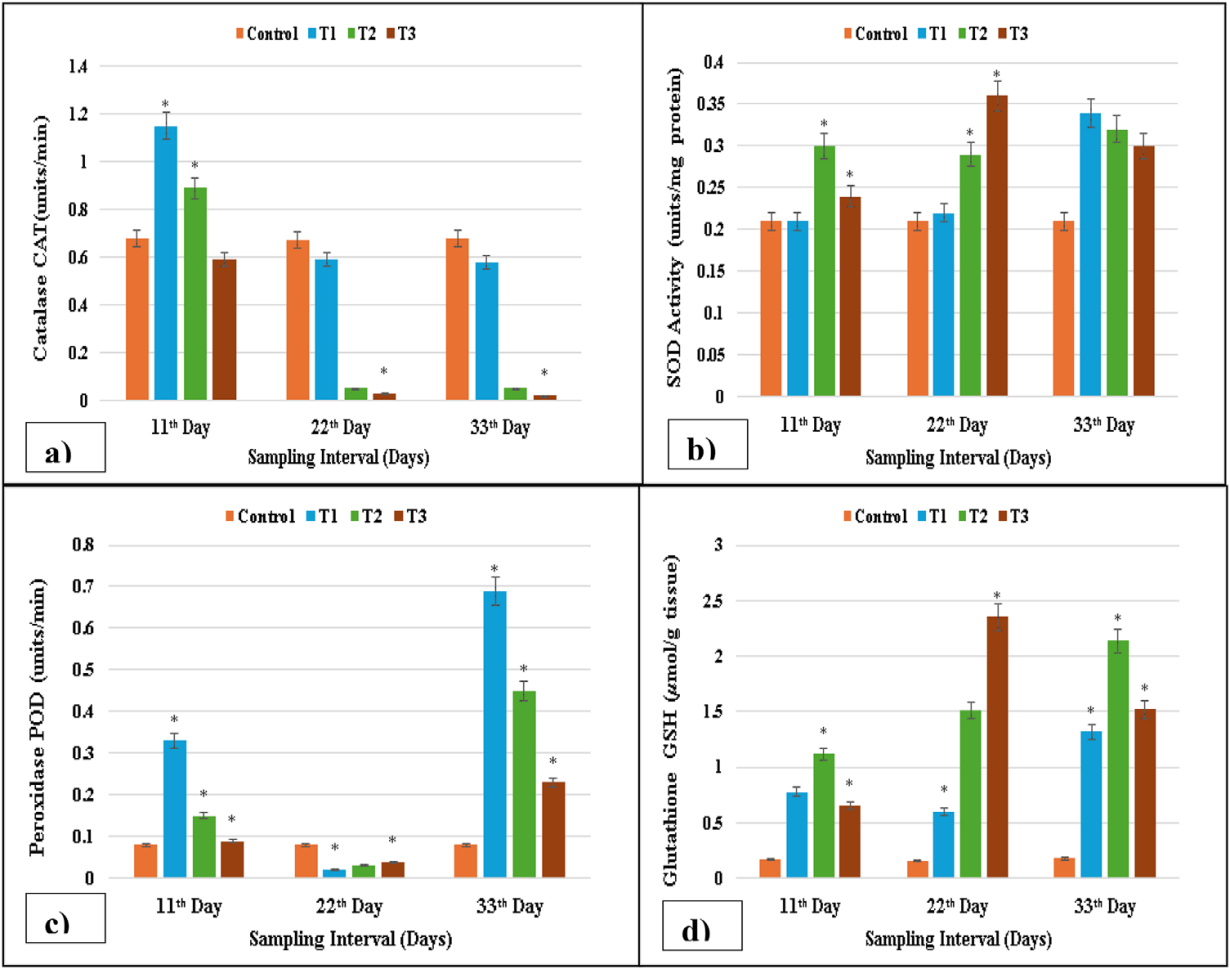
Antioxidant enzyme activity: (a) CAT activity decreased at maximum concentration on Days 22 and 33; (b) SOD activity increased at maximum concentration at 11, 22 and 33 days; (c) POD activity was reduced at 11 and 22 days, whereas it increased at 33 days at lower concentration and decreased at higher concentration; (d) GSH activity increased at maximum concentration on 22 and 33 days in isolated cells of the liver of lufenuron‐treated and control fish. SOD, superoxide dismutase. * indicates significantly different at *p* < 0.05.

On experimental days 11, 22 and 33, fish in T1 (2 µg/L), T2 (3 µg/L) and T3 (4 µg/L) groups treated with lufenuron exhibited changes in levels of antioxidant enzymes GSH, POD, SOD and CAT in the liver. The outcomes obtained from this research showed that the SOD and GSH contents of the treatment groups were higher as compared to the control group. At the same time, the POD and the activity of CAT were significantly (*p* < 0.05) higher in the liver of lufenuron‐treated *C. idella* as compared to the control group, as shown in Figure 2.

### 3.4 Antioxidant Enzymes in Kidney

**FIGURE 3:**
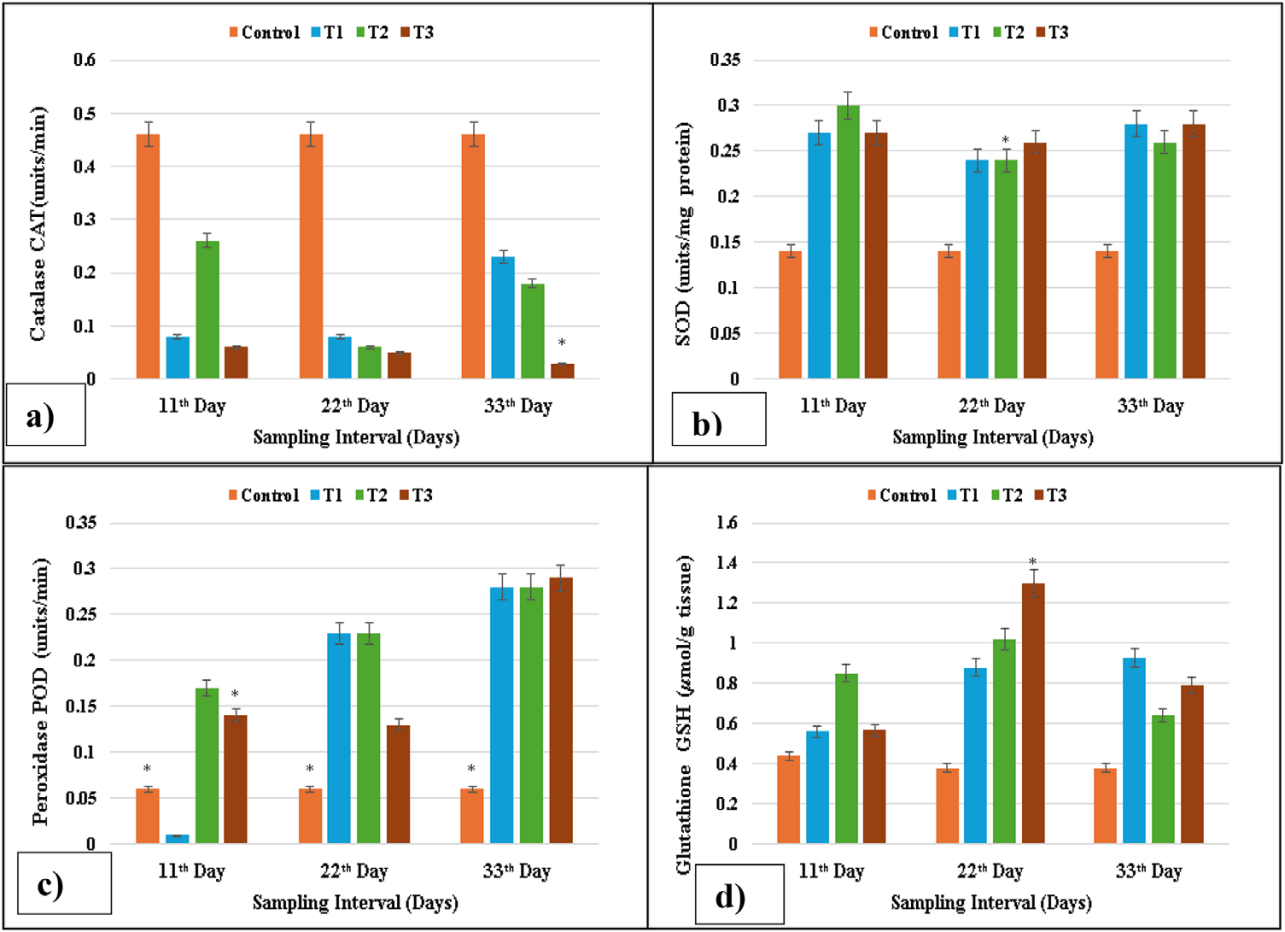
Antioxidant enzyme activity: (a) CAT activity reduced at higher concentrations on Days 11, 22 and 33; (b) SOD activity increased at higher concentrations at each sampling; (c) POD activity increased at higher concentrations on Days 22 and 33, whereas it was reduced at the maximum concentration on Day 22; (d) GSH activity enhanced at the higher concentration on Day 22, whereas it was reduced on Day 33 in isolated cells of kidneys of lufenuron‐treated and control fish. SOD, superoxide dismutase. * indicates significantly different at *p* < 0.05.

The fishes belonging to group T1 treated with 2 µg/L lufenuron after Days 11, 22 and 33 had a significant alteration in levels of POD, SOD, CAT and GSH levels. The SOD and GSH contents of the treatment groups were higher as compared to the control group, whereas the activity of POD was significantly (*p* < 0.05) lower in the kidney of grass carp. The activity of CAT was significantly (*p* < 0.05) increased in the kidney of exposed *C. idella* as compared to the control, as shown in Figure 3.

### 3.5 Antioxidant Enzymes in Heart

**FIGURE 4:**
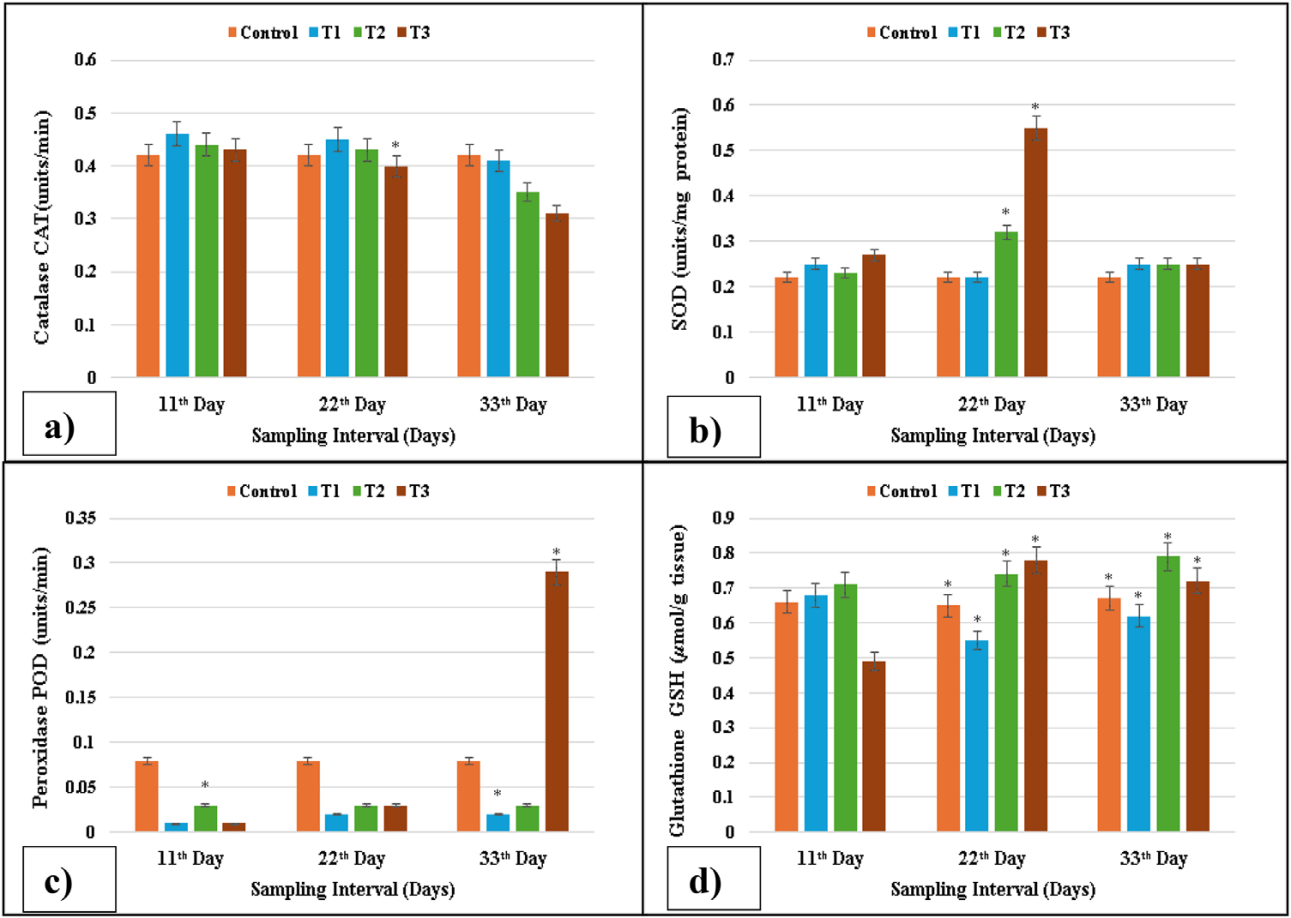
The antioxidant enzyme activity: (a) CAT activity reduced at 33 days; (b) SOD activity increased on Day 33 at higher concentration; (c) POD activity reduced on Days 11 and 22, whereas it was increased on Day 33 at higher concentration; (d) GSH activity shows little difference at each concentration in isolated cells of the kidney of lufenuron‐treated and control fish. SOD, superoxide dismutase. * indicates significantly different at *p* < 0.05.

On trial days 11, 22 and 33, fish in the T1 (2 µg/L) group treated with lufenuron showed significant changes in levels of the antioxidant enzymes in the heart. From this research, the results showed that the SOD and GSH contents of the treatment groups were higher as compared to the control group, whereas the activity of POD was significantly (*p* < 0.05) increased in the heart as compared to the control. However, CAT activity was significantly (*p* < 0.05) decreased in the heart. Specifically, in the T2 and T3 groups treated with 3 and 4 µg/L lufenurons, respectively, there was a substantial decrease, as shown in Figure 4.

### 3.6 Antioxidant Enzymes in Brain

**FIGURE 5:**
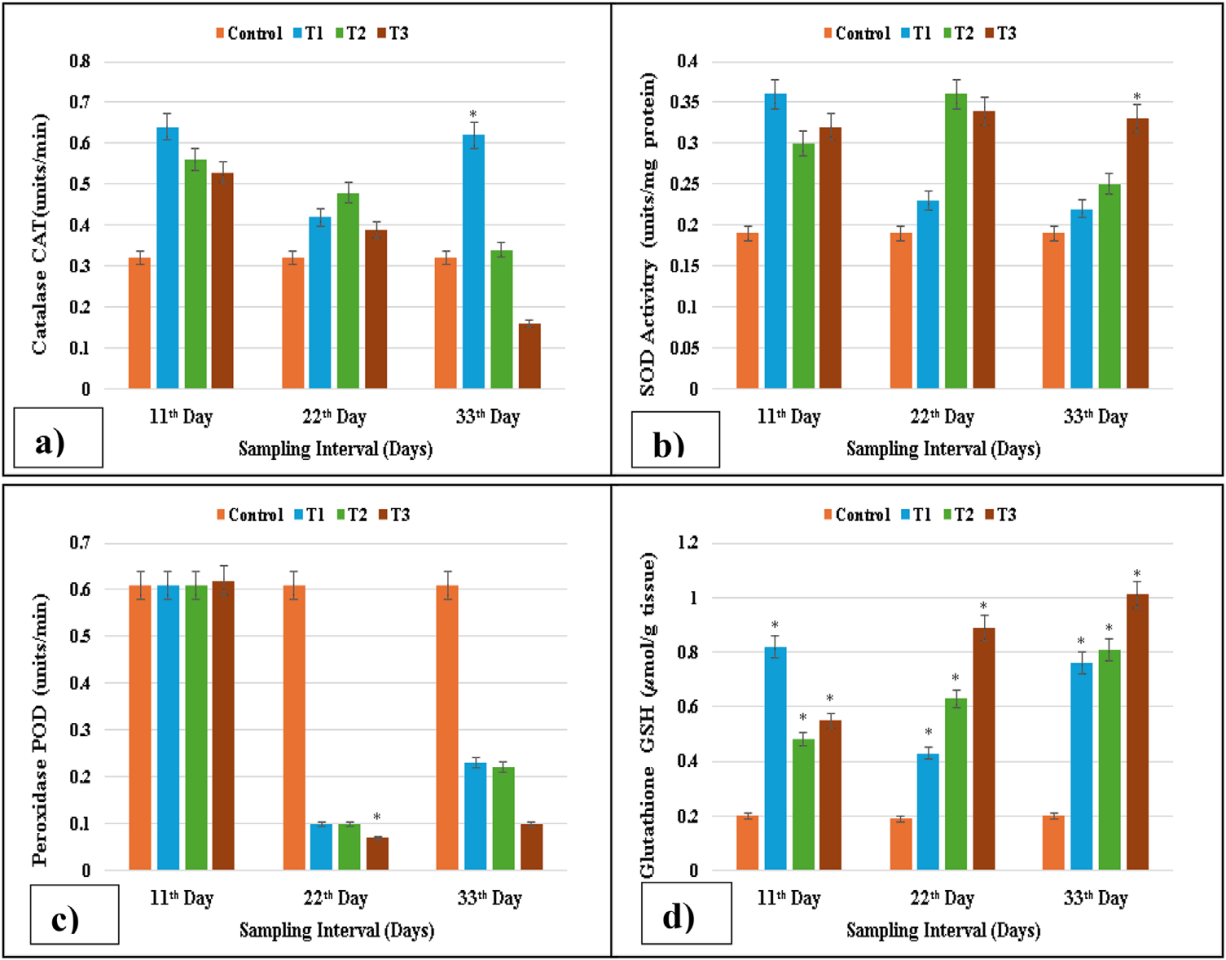
Antioxidant enzyme activity: (a) CAT activity reduced on Day 33 at maximum concentration; (b) SOD activity increased on Day 33 at higher concentration; (c) POD activity suddenly decreased on Days 22 and 33 at higher concentrations; (d) GSH activity increased on Days 22 and 33 at maximum concentration in isolated cells of the brains of lufenuron‐treated and control fish. SOD, superoxide dismutase. * indicates significantly different at *p* < 0.05.

The first treated with different concentrations of lufenuron showed various changes in antioxidant enzyme activities. The results showed that the SOD and GSH contents of the treatment groups were higher as compared to the control group, whereas the POD action was increased in the brain as compared to the control. However, CAT activity was significantly (*p* < 0.05) decreased in the brain, as shown in Figure 5.

## 4 Discussion

The current study revealed that lufenuron exposure significantly affected the antioxidant defence system in different organs of *C. idella*. Antioxidant enzymes, such as SOD, CAT, POD and GSH, play essential roles in protecting cells from oxidative damage by scavenging reactive oxygen species (ROS). These enzymes serve as the first line of defence against oxidative stress, and alterations in their activities indicate changes in the cellular redox status (Clempus and Griendling 2006; Gravato et al. 2006).

Our findings demonstrated that SOD and GSH activities were consistently elevated in all organs of lufenuron‐treated fish compared to the control group. This suggests that the pesticide induced oxidative stress and triggered a compensatory upregulation of antioxidant defences. SOD catalyses the conversion of superoxide radicals to hydrogen peroxide, which is subsequently decomposed by CAT and POD, whereas GSH acts as a non‐enzymatic antioxidant by directly neutralizing ROS and maintaining cellular redox balance. Similar increases in antioxidant enzyme activity under pesticide stress have been reported in several fish species, including *Danio rerio*, *Oreochromis mossambicus* and *Clarias gariepinus*.

The organ‐specific analysis in the present study highlighted differential responses to lufenuron exposure. In gills, SOD and CAT activities were elevated, whereas POD declined significantly. As gills are in constant contact with the external environment and serve as a primary site for xenobiotic uptake, they are often the first organ to show biochemical responses to toxicants (Rajinder and Kaur 2014). The reduction of POD in gills may indicate that hydrogen peroxide detoxification relied more on CAT activity under pesticide stress.

The liver, being the central organ for metabolism and detoxification, showed significant elevation of all four enzymes, reflecting enhanced activity of both enzymatic and non‐enzymatic antioxidant systems. This strong response can be attributed to increased metabolic demand for pesticide detoxification, which generates ROS as by‐products. Comparable findings were observed in *Carassius auratus* and *Prochilodus lineatus* exposed to other pesticides, where the liver showed marked induction of antioxidant enzymes.

In the kidney, activities of SOD, CAT and GSH increased, whereas POD activity was reduced. As the kidney is involved in excretion and osmoregulation, increased enzyme activity reflects an adaptive response to lufenuron‐induced stress, whereas the reduction in POD may be due to enzyme inhibition or tissue‐specific vulnerability. Similarly, in the heart and brain, SOD, POD and GSH were upregulated, but CAT activity declined significantly. The decline in CAT activity in these sensitive tissues may lead to insufficient hydrogen peroxide breakdown, increasing oxidative pressure and rendering these organs more susceptible to cellular damage. Previous studies in *Pangasius hypophthalmus* and *D. rerio* reported similar organ‐specific reductions in CAT activity under pesticide exposure, indicating that CAT inhibition is a consistent marker of pesticide‐induced oxidative stress (Sutha et al. 2022).

The variability of antioxidant enzyme responses across organs in this study highlights the complex interplay between ROS generation, antioxidant defence and tissue function. Although some organs, like the liver and gills, showed strong compensatory responses, others, such as the brain and heart, exhibited reduced CAT activity, suggesting tissue‐specific susceptibility. Such organ‐dependent patterns of antioxidant enzyme alteration have also been described in fish exposed to different classes of pesticides (Lukaszewicz‐Hussain 2010).

In addition to biochemical changes, notable behavioural alterations were observed in lufenuron‐exposed fish, including erratic swimming, excessive mucus secretion, restlessness and reduced feeding. These symptoms are commonly associated with neurotoxic and oxidative effects of pesticides on fish physiology (Nwani et al. 2010). The combination of behavioural and biochemical responses provides strong evidence that lufenuron induces significant stress in *C. idella*, potentially impairing its survival and performance in aquaculture systems.

Taken together, the findings of this study confirm that antioxidant enzymes are sensitive biomarkers for pesticide‐induced oxidative stress. Lufenuron exposure not only altered antioxidant defences but also revealed organ‐specific vulnerabilities, especially in gills, brain and heart, which are directly involved in vital physiological functions. The consistent elevation of SOD and GSH across all tissues underscores their critical role in counteracting oxidative stress, whereas the organ‐specific reduction of CAT suggests potential long‐term risks of oxidative damage. These results are in agreement with earlier studies reporting that pesticide exposure disrupts oxidative balance and may compromise fish health at biochemical, physiological and behavioural levels (Nwani et al. 2010; Ghelichpour et al. 2019).

Overall, the current study provides important ecotoxicological evidence on the oxidative impact of lufenuron in freshwater fish. The results not only contribute to understanding the mechanistic basis of lufenuron toxicity but also emphasize the need to regulate and monitor pesticide residues in aquatic environments. Such measures are crucial to ensure the sustainability of aquaculture practices and the protection of aquatic biodiversity.

## 5 Conclusions and Future Works

Lufenuron exposure induced significant oxidative stress in *C. idella*, as reflected by organ‐specific alterations in antioxidant enzymes. The liver and gills showed strong compensatory responses, whereas reduced CAT activity in the heart and brain suggested higher vulnerability to oxidative damage. These findings confirm that antioxidant enzymes serve as reliable biomarkers of pesticide toxicity and highlight the ecological risk of lufenuron contamination in aquatic environments.

## Author Contributions

Maria Saeed Khan conducted the research and prepared the manuscript. Habiba Jamil collected and analysed the data. Abdul Ghaffar and Qudrat Ullah supervised the study and critically reviewed the manuscript. Ibrahm A. Alhidary provided resources and funding support. Muhammad Israr contributed to manuscript drafting and revision.

## Conflicts of Interest

The authors declare no conflicts of interest.

## Peer Review

The peer review history for this article is available at https://www.webofscience.com/api/gateway/wos/peer‐review/10.1002/vms3.70626.

## Data Availability

The data used to support the outcomes of this study are available on request from the corresponding author.
